# Biallelic *SLC13A1* loss-of-function variants result in impaired sulfate transport and skeletal phenotypes, including short stature, scoliosis, and skeletal dysplasia

**DOI:** 10.1016/j.gimo.2024.101958

**Published:** 2024-12-26

**Authors:** Christina G. Tise, Katie Ashton, Lachlan de Hayr, Kun-Di Lee, Omkar L. Patkar, Emma Krzesinski, Jennifer A. Bassetti, Erin M. Carter, Cathleen Raggio, Andreas Zankl, Anas M. Khanshour, Kristhen N. Atala, Jonathan J. Rios, Carol A. Wise, Ying Zhu, Futao Zhang, Tony Roscioli, Michael Buckley, Robert J. Harvey, Paul A. Dawson

**Affiliations:** 1 Division of Medical Genetics, Department of Pediatrics, Lucile Packard Children’s Hospital and Stanford University, Stanford, CA; 2 New South Wales Health Pathology, Randwick Genomics, Prince of Wales Hospital, New South Wales, Australia; 3 School of Health, University of the Sunshine Coast, Maroochydore, Queensland, Australia; 4 National PTSD Research Centre, Thompson Institute, University of the Sunshine Coast, Birtinya, QLD, Australia; 5 Mater Research Institute, University of Queensland, Brisbane, QLD, Australia; 6 Monash Genetics, Monash Medical Centre, Melbourne, VIC, Australia; 7 Department of Paediatrics, Monash University, Clayton, VIC, Australia; 8 Division of Medical Genetics, Department of Pediatrics, Weill Cornell Medicine, New York, NY; 9 Hospital for Special Surgery, New York, NY; 10 Department of Clinical Genetics, The Children’s Hospital at Westmead, Westmead, New South Wales, Australia; 11 Faculty of Medicine and Health, The University of Sydney, Camperdown, New South Wales, Australia; 12 Garvan Institute of Medical Research, Darlinghurst, New South Wales, Australia; 13 Center for Translational Research, Scottish Rite for Children, Dallas, TX; 14 Department of Pathology, University of Texas Southwestern Medical Center, Dallas, TX; 15 McDermott Center for Human Growth and Development and the Departments of Orthopaedic Surgery and Pediatrics, University of Texas Southwestern Medical Center, Dallas, TX; 16 Neuroscience Research Australia (NeuRA), Prince of Wales Clinical School, University of New South Wales, Sydney, Australia

**Keywords:** Hyposulfatemia, Scoliosis, Short stature, Skeletal dysplasia, Sulfate transporter

## Abstract

**Purpose:**

Sulfate is vital for many physiological processes, including the structural and functional maintenance of macromolecules and formation of sulfur-containing compounds essential for cartilage and bone development. SLC13A1 is a sodium-sulfate cotransporter primarily expressed in the kidney, where it mediates sulfate reabsorption and maintenance of circulating sulfate levels. In this study, we characterized the clinical, biochemical, and functional impact of biallelic *SLC13A1* nonsense and/or missense variants in individuals presenting with a skeletal phenotype.

**Methods:**

Probands were identified by exome or genome sequencing and GeneMatcher. Sulfate levels were quantified using ion chromatography. *SLC13A1* missense variants p.(Arg237Cys), p.(Gly448Asp), p.(Leu516Pro), and p.(Tyr582His) were characterized using bioinformatics, molecular modeling, and [^35^S]-sulfate uptake assays in Madin-Darby canine kidney cells.

**Results:**

All probands presented with concern for short stature and were found to have scoliosis and/or skeletal dysplasia. A reduction in plasma sulfate level and/or increase in urinary sulfate excretion was detected in 2 of 2 probands evaluated. Functional studies were consistent with *SLC13A1* variants resulting in the complete loss of sulfate transport activity.

**Conclusion:**

Biallelic loss-of-function variants in *SLC13A1* are a novel cause of skeletal phenotypes in humans with a measurable biomarker. Sulfate measurements should be considered in the clinical interpretation of variants identified in *SLC13A1*.

## Introduction

Sulfate (SO_4_^2-^) is vital for many physiological processes, including structural and functional maintenance of many macromolecules and the formation of hundreds of sulfur-containing compounds.^1^^,^^2^ Inherited metabolic disorders (IMDs) in which disturbed sulfate homeostasis is the underlying mechanism of disease often result in extracellular matrix abnormalities of connective tissues and/or abnormal accumulations of glycosaminoglycans.^1^ Such IMDs are due to pathogenic variants in genes involved in maintaining the physiological ratio of sulfated to nonsulfated molecules. Specifically, the role of sulfate in skeletal growth and development is exemplified by the various abnormal bone and cartilage phenotypes exhibited by individuals with certain forms of skeletal dysplasias or mucopolysaccharidoses.^1^^,^^3^^,^^4^

Sulfate transporters in the kidney are particularly important in maintaining the circulating sulfate level, which provides a reservoir of sulfate for uptake into tissues throughout the body.^1^^,^^3^^,^^5^ The typical fractional excretion index of sulfate is 0.17 to 0.34,^6^^,^^7^ with normal serum sulfate levels ranging between 0.3 to 0.5 mM, making sulfate the fourth most abundant anion in human circulation.^1^^,^^2^ The sulfate transporter, solute carrier family 13, member 1 (*SLC13A1*; HGNC: 10916; NCBI Gene ID: 6561), is primarily expressed in the renal proximal tubule where it mediates the first step of sulfate reabsorption.^8^ Consistent with the critical role of SLC13A1 in sulfate homeostasis, 2 rare, unlinked, heterozygous nonsense variants in the corresponding gene, *SLC13A1,* NM_022444.4:c.34C>T p.(Arg12∗) and NM_022444.4:c.144G>A p.(Trp48∗), have previously been associated with a 27% reduction in serum sulfate levels (0.25 vs 0.36 mM in controls), whereas individuals homozygous or compound heterozygous for these alleles were not identified.^9^

The *Slc13a1* knockout mouse model exhibits hyposulfatemia and impaired growth, along with increased susceptibility to acetaminophen-induced hepatotoxicity and several other endocrine, neurodevelopmental, and reproductive phenotypes.^10^, ^11^, ^12^, ^13^, ^14^ Additionally, dogs and sheep found to have naturally occurring homozygous loss-of-function (LOF) variants in *Slc13a1* exhibit hyposulfatemia and a form of osteochondrodysplasia characterized by growth restriction and angular deformities of the forelimbs.^15^^,^^16^ More recently, a child with spondyloepimetaphyseal dysplasia (SEMD) was found to be homozygous for the p.(Arg12∗) variant and exhibited hyposulfatemia on further evaluation.^17^

Given the biochemical, skeletal, and cartilage phenotypes described to date, we hypothesized that humans with biallelic LOF variants in *SLC13A1* may present with a skeletal dysplasia phenotype and markedly decreased plasma sulfate and/or increased urinary sulfate excretion due to disruption of sulfate transport via SLC13A1. Through the use of exome or genome sequencing and GeneMatcher (https://genematcher.org), 5 children from 4 families with biallelic variants in *SLC13A1* and a skeletal dysplasia phenotype were identified. This allowed further assessment of the clinical and functional impact of these variants using bioinformatics, molecular modeling, and [^35^S]-sulfate uptake assays in Madin-Darby canine kidney (MDCK) cells.

## Materials and Methods

### Cases and data collection

This investigation included 5 children with abnormal skeletal findings and biallelic variants in *SLC13A1* identified exome or genome sequencing. All clinical data were collected directly from the clinicians providing care to the participants and their families and from chart review of clinical evaluation including results of genetic, radiological, and analyte testing.

### Sulfate measurement in plasma and urine

Blood samples were collected, and plasma was isolated and stored at −20 °C. Urine samples were collected around the time of blood collection and stored at −20 °C. Plasma and urine sample sulfate concentrations were measured using ion chromatography with suppressed conductivity detection using a Dionex ICS2000, as described previously.^18^ Urinary and plasma creatinine were measured using a Vitros 5.1 FS chemistry analyzer. The fraction excretion index of sulfate was calculated using the formula: (urinary sulfate mM × plasma creatinine mM) / (plasma sulfate mM × urinary creatinine mM).

### Bioinformatics and genotyping

In silico scores for *SLC13A1* missense variants were calculated using the VarCards D:A algorithm, ClinPred, CADD, and REVEL; missense variants predicted to be pathogenic or damaging by ≥1 bioinformatics tools were included in molecular modeling and functional experiments. Proband and parental DNA sample quality assessment, library preparation, exome or genome sequencing, and data processing were performed based on each site’s local clinical and/or research protocol. Sequencing was performed using DNA isolated from blood samples. All variants were validated using standard Sanger sequencing and/or in a Clinical Laboratory Improvement Amendment-certified laboratory or National Association of Testing Authorities-accredited laboratory. The human sequence variants in this manuscript are described using the reference sequences NM_022444.4 (transcript) and NP_071889.2 (protein). The reference genome build GRCh38/hg38 was used for alignments.

### Molecular modeling

A homology model of human SLC13A1 was constructed using SwissModel, based on the structure of a related family member SLC13A5, which encodes a NaCT-Citrate complex (RCSB PDB: 7JSK)^19^ in the inward-facing confirmation. The impacts of *SLC13A1* missense variants were examined using ChimeraX^20^ using the Swapaa command and the Dunbrack backbone-dependent rotamer library.^21^

### Generation of mEmerald-*SLC13A1* expression constructs and MDCK cell transfection

Human *SLC13A1* cDNA was amplified from human placenta first-strand cDNA (Takara Bio) using Platinum SuperFi PCR Master Mix (ThermoFisher) and cloned into pmEmerald-C1 using the NEBuilder HiFi DNA Assembly Master Mix (NEB) and primers listed in Supplemental Table 1. Missense variants were introduced into the *SLC13A1* cDNA with a 2-fragment mutagenesis protocol^22^ using NEBuilder HiFi DNA assembly, using primers in the origin of replication (ColE1F and ColE1R), and overlapping the desired variant in the *SLC13A1* cDNA (Supplemental Table 1). Nucleotide sequences of *SLC13A1* variants were verified by Sanger sequencing. MDCK cells^23^ were transfected using Lipofectamine 2000 (Invitrogen) with pmEmerald-C1 (empty vector) or pmEmerald-*SLC13A1* wild-type and mutant constructs. Cells were cultured in Dulbecco’s Modified Eagle Medium (Gibco) containing 10% fetal bovine serum and supplemented with 0.8 mg/ml of Geneticin (Life Technologies) to select stably transfected cell lines.

### Radiotracer [^35^S]-sulfate uptake into cultured cells

MDCK cells are an established functional model for analyzing SLC13 family member transporter function because the cells show polarity and both sulfate transport function and targeting to apical and basolateral membranes can be monitored.^23^ Stably transfected MDCK cells at approximately 95% confluency in 12-well tissue culture plates were rinsed twice with 1-ml assay buffer (137-mM NaCl, 5.3-mM KCl, 2.8-mM CaCl_2_, 1.2-mM MgCl_2_, 20-mM HEPES/Tris pH 7.4) and 0.3 ml of assay buffer containing 0.5 mM of K_2_SO_4_ and 2 μCi/ml of [^35^S]-sulfate (1,050-1,600 Ci/mmol, Perkin Elmer) were added and incubated at room temperature for 15 minutes. Cells were washed 3 times in ice-cold assay buffer (137-mM NaCl and 10-mM Tris/HCl pH 7.4) and then incubated in 500 μl of lysis buffer (1% Triton X-100) for 1 hour. 150 μl of lysate and 1.5 ml of scintillation fluid (Ultima Gold liquid scintillation cocktail, Perkin Elmer) were added to a scintillation vial and cpm was measured using a MicroBeta TriLux Liquid Scintillation Counter (PerkinElmer). Sulfate uptake for SLC13A1 missense variants was compared with control SLC13A1 using one-way ANOVA followed by a Dunnett’s multiple comparison test.

### Confocal microscopy

Stably transfected MDCK cells were grown to confluency on glass coverslips, fixed for 20 minutes with 4% (w/v) paraformaldehyde, then rinsed in PBS. Cells were permeabilized with 0.25% (v/v) Triton X-100, rinsed in PBS, and incubated with phalloidin (1:500 dilution, Sigma-Aldrich) to stain for actin filament and with DAPI to stain the nucleus (1:10,000 dilution, Sigma-Aldrich). Cells were washed in PBS and mounted on glass slides using Mowiol (Calbiochem). Confocal images were obtained using a Zeiss LSM 510 Meta confocal microscope.

## Results

### Case presentations

**Figure 1:**
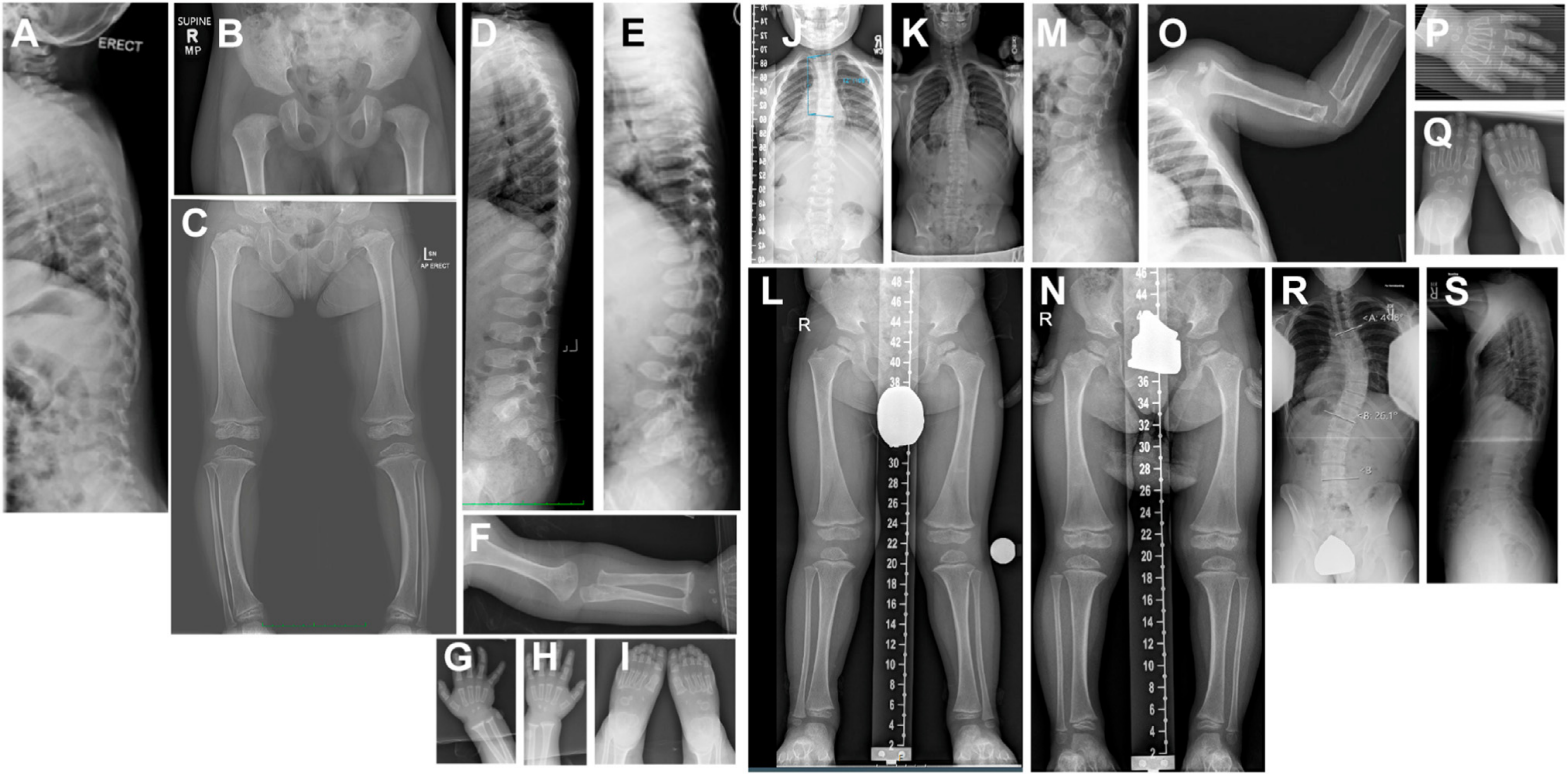
**Radiographic images of probands with biallelic *SLC13A1* loss-of-function variants.** A and B. Proband 1: mild anterior beaking of the lumbar vertebrae (A) and delayed ossification of the proximal capital femoral epiphyses with a stippled appearance at 2 years old (B). C and D. Proband 2 at 3 years old: tibial bowing and spondyloepiphyseal dysplasia with small and fragmented proximal capital femoral epiphyses (C) and wedge-shaped vertebral bodies (D). E-I. Proband 3 at 14 months old: thoracic kyphosis and lumbar lordosis with platyspondyly, wedging, and anterior beaking of thoracic and lumbar vertebrae (E), nonossification of the proximal humeral epiphysis with shortening of tubular bones and widened metaphyses (F-H), irregular and sclerotic capitate and hamate (G and H), and normal tarsal bones (I). J and K. Proband 3: 12° thoracic curve at 4 years old (J) with progression to S-shaped scoliosis with 28° upper thoracic (T3-T8), 19° lower thoracolumbar (T10-L3), and 11° lumbar curve at 13 years old (K). L. Proband 3 at 3 years old: valgus of bilateral knees with foreshortening of proximal femoral metaphyses and widening of the femoral and tibial metaphyses. M-P. Proband 4 at 14 months old: ovoid and bullet shaped vertebral bodies with anterior wedging (M), shorted tubular bones with widened metaphyses and fragmented proximal epiphyses (N-P), and normal appearing carpal and tarsal bones (O and P). Q. Proband 4 at 2 years old: slightly flared proximal femoral metaphyses bilaterally with relative foreshortening. R and S. Proband 5: 41° and 44° dextroconvex thoracic curve at 14 years old and 17 years old, respectively, with levoconvex lumbar scoliosis with diminished vertebral body height of multiple mid and lower thoracic bodies.

Growth parameters, clinical features, identified *SLC13A1* variants, sulfate measurements, and radiographic interpretations for each proband and family are summarized in Table 1. Radiographic images for each proband are shown in Figure 1 and Supplemental Figures. For all probands, no other variants with a higher likelihood of being disease causing were identified.

**Table 1:** Clinical features of individuals with biallelic loss-of-function variants in *SLC13A1*

Family	Individual (Current Age, Sex)	*SLC13A1* Genotype	Height	Weight	Orthopedic and Radiologic Features	Additional Features
A	Proband 1 3 yo M	p.(Arg12∗) (paternal) p.(Gly448Asp) (maternal)	<−2 SD Normal upper:lower body segment ratio	N/A	9 mo: delayed ossification of the proximal capital femoral epiphyses with a stippled appearance (Figure 1B) 2 yo: mild anterior beaking of the lumbar vertebrae (Figure 1A)	Mild dysmorphic facial features (bilateral epicanthal folds, a flattened nasal bridge and a short and anteverted nose), tapered fingers, and deep-set nails of halluces. Urinary sulfate/creatinine ratio (ref 0.5-6.0) • Proband: 10.96 • Father: 1.71 • Mother 3.81
B	Proband 2 12 yo F	p.(Tyr582His), homozygous with confirmed biparental inheritance	137.7 cm (3rd %ile, −1.85 SD)	42 kg (51st %ile, 0.02 SD)	3 yo: tibial bowing and spondyloepiphyseal dysplasia with small and fragmented proximal capital femoral epiphyses (Figure 1C) and wedge-shaped vertebral bodies (Figure 1D) 11 yo: improved leg bowing, development of mild scoliosis	Plasma sulfate = 0.107 mM (ref 0.3-0.5 mM) Fraction excretion index of sulfate = 0.038 (ref 0.17-0.34)
C	Proband 3 13 yo M	p.(Trp48∗) (paternal) p.(Leu516Pro) (maternal)	129.6 cm (5th %ile, −1.66 SD)	33 kg (47th %ile, −0.06 SD)	14 mo: exaggerated thoracic kyphosis and lumbar lordosis (Figure 1E), anterior beaking of several thoracic and lumbar vertebral bodies (Figure 1E), non-ossification of the proximal humeral epiphyses, flaring of the distal and proximal humeral metaphyses and distal radial and ulnar metaphyses (Figure 1F-H), irregular and sclerotic capitate and hamate (Figure 1G and H); unremarkable skull 3 yo: small appearing pelvis relative to proximal femora, short and broad femoral necks, nonossification of the capital femoral epiphyses, proximal and distal flaring of the femoral and tibial metaphyses, small and irregular epiphyses of the knees (Figure 1L) 4 yo: 12° thoracic curve (Figure 1J) 13 yo: progression to S-shaped thoracolumbar scoliosis with 28° thoracic curve (Figure 1K)	Prematurity, type 1 Chiari malformation with resolved ventriculomegaly, strabismus (resolved), pectus carinatum, atrial septal defect s/p repair, broad toes
			134.4 cm (20th %ile, −0.85 SD)	36.5 kg (60th %ile, 0.28 SD)	14 mo: ovoid vertebral bodies with anterior wedging, accentuated, lumbosacral lordosis (Figure 1M), shorted tubular bones with widened metaphyses and fragmented proximal epiphyses (Figure 1N) 2 yo: broad iliac bones, short and broad femoral necks, short tibiae (Figure 1Q)	Prematurity, broad thumbs, 2,3 toe syndactyly, short and broad halluces
	Proband 4 13 yo F					
D	Proband 5 17 yo M	p.(Trp48∗), homozygous with confirmed biparental inheritance	157.3 cm (<2nd %ile, −2.13 SD)	59 kg (39th %ile, −0.27 SD)	14 yo: 41° dextroconvex thoracic curve and levoconvex lumbar scoliosis (Figure 1R) 17 yo: 44°dextroconvex thoracic curve and levoconvex lumbar scoliosis; normal bone mineralization; diminished vertebral body height of multiple mid and lower thoracic bodies; endplates are concave (Figure 1S)	Mild to moderate neurocognitive impairment affecting sensory, motor, attention, and subcortical abilities, atypical developmental disorder and Asperger's disorder, atypical mild attentional disorder, generalized anxiety disorder, social anxiety disorder, masked depression, obsessive compulsive personality traits

Proband 1 is a 3-year-old male who presented at 2 years of age with increasing hip pain and a history of bilateral developmental hip dysplasia requiring bracing until 9 months of age. The proband is the first-born child of healthy, nonconsanguineous parents of European ancestry; the mother was pregnant with her second child at the time of the initial evaluation. Physical examination was notable for a normal gait and proportionate short stature (*z-*score < −2 SD) with a normal upper:lower body segment ratio. He had mild dysmorphic facial features, including bilateral epicanthal folds, a flattened nasal bridge, and a short and anteverted nose. He had tapered fingers, and the nails of his halluces were deep set. Serial pelvic X-rays from age 9 months showed delayed and stippled femoral ossifications consistent with the diagnosis of multiple epiphyseal dysplasia. Lateral spine X-ray performed at 2 years of age demonstrated mild anterior beaking of the lumbar vertebra (Figure 1A and B). Trio exome sequencing identified biallelic variants in *SLC13A1*: paternally inherited NM_022444.4:c.34C>T p.(Arg12∗) and maternally inherited NM_022444.4:c.1343G>A p.(Gly448Asp). Copy-number variant (CNV) analysis was performed using exome sequencing data and did not detect any clinically relevant CNVs. Urine sulfate levels were measured in the proband and parents, demonstrating elevated urinary sulfate excretion in the proband (sulfate/creatinine ratio 10.96; reference: 0.5-6.0) and normal urinary sulfate excretion in the parents (father: 1.71; mother 3.81). Blood from the trio was collected for plasma sulfate quantification. However, measurements were not performed because of inadequate specimen transportation to the laboratory.

Proband 2 is a female and the first child of healthy, nonconsanguineous parents of Asian ancestry. She was born at term after an uncomplicated pregnancy and delivery, although birth measurements were unavailable. Around 14 months of age, she began walking and was noted to have bowed legs. Orthopedic evaluation at 3 years of age revealed tibial bowing and spondyloepiphyseal dysplasia with small and fragmented proximal capital femoral epiphyses and wedge-shaped vertebral bodies (Figure 1C and D). When she was reevaluated at 11 years of age, her leg bowing had improved spontaneously, but she had developed mild scoliosis. Her height has been proportionate and has continually tracked along the lower 2nd SD for age, whereas her weight has increased from the 15th percentile to the 50th percentile. Most recent measurements at 12 years of age were 137.7 cm (3rd percentile, *z* = −1.85) and 42 kg (51st percentile, *z* = 0.02). Trio genome sequencing did not identify an etiology within a known disease gene. However, a targeted search for variants in *SLC13A1* identified a homozygous missense variant, NM_022444.4:c.1744T>C p.(Tyr582His), with biallelic inheritance. Sulfate measurements in the proband showed an increased fraction excretion index of sulfate of 0.38 (reference: 0.17-0.34), and a reduced plasma sulfate of 0.107 mM (reference: 0.3-0.5 mM; mean among individuals with heterozygous *SLC13A1* nonsense variants: 0.25 mM), consistent with this variant representing a LOF allele of similar effect size to that of the nonsense variants.

Proband 3 (male) and proband 4 (female) are fraternal twins who presented for Medical Genetics evaluation after X-rays performed on proband 3 at 10 months of age for a pectus carinatum deformity demonstrated abnormal vertebrae. The twins were conceived via in vitro fertilization and born via spontaneous vaginal delivery at 34-weeks gestation to healthy, nonconsanguineous parents. Proband 3 weighed 2 kg and was 42.5 cm long at birth, whereas proband 4 weighed 1.9 kg and was 46 cm long. Proband 3 was admitted to the neonatal intensive care unit for 23 days for respiratory issues, and past medical history was notable for a type 1 Chiari malformation with resolved ventriculomegaly of the 3rd and lateral ventricles, an atrial septal defect status post-surgical repair, idiopathic thrombocytopenia, and strabismus treated with patching and glasses. Proband 4 required neonatal intensive care unit admission for 10 days because of prematurity.

Several skeletal surveys were performed on proband 3 (Figure 1E-L), which showed exaggerated thoracic kyphosis and exaggerated lumbar lordosis, anterior beaking of several thoracic and lumbar vertebral bodies, short and broad femoral necks, and proximal and distal flaring of the femoral and tibial metaphyses, consistent with SEMD, interpreted as “multiple skeletal abnormalities most consistent with Kniest dysplasia.” His physical examination at the time of the evaluation was significant for normal gait, mild pectus deformity, subtle disproportion of the limbs, and broad toes. Given these findings, proband 4 also underwent a skeletal survey at 14 months and 2 years of age (Figure 1M-Q), which showed ovoid vertebral with anterior wedging, broad and short femoral necks, and widened metaphyses of tubular bones consistent with SEMD, interpreted as “numerous osseous abnormalities compatible with Kniest syndrome.” Her physical examination was significant for normal gait, broad thumbs, 2,3 toe syndactyly, and short and broad halluces. Urine glycosaminoglycan analysis was within normal limits for proband 3, and *COL2A1* sequencing did not detect any variants in either twin.

At 10 years of age, the twins returned for evaluation of shorter than expected proportionate stature (proband 3 height 129.6 cm, 5th percentile, *z* = −1.66; proband 4 134.4 cm, 20th percentile, *z* = −0.85) with prior height trajectory of proband 3 and proband 4 tracking along the 1st-2nd and 5th-10th percentile, respectively. The twins are the only children of their parents who are both healthy and of normal stature (mother: 5’3.5”, father: 6’0”, mid-parental height: 50th percentile). Quad exome sequencing identified biallelic variants in *SLC13A1* in both twins: paternally inherited NM_022444.4:c.1547T>C p.(Leu516Pro) and maternally inherited NM_022444.4: c.144G>A p.(Trp48∗). CNV analysis was performed using exome sequencing data and did not detect any clinically relevant CNVs of 3 or more exons in the data for either proband. Blood and urine sulfate levels could not be obtained.

Proband 5 is a male and only child of healthy, nonconsanguineous parents who presented with concern for scoliosis at 14 years of age. Pregnancy and birth were uncomplicated and there was no history of prior surgery. Growth parameters were notable for proportionate short stature (157.3 cm, 1.7th percentile, *z* = −2.13) with normal weight (59 kg, 39th percentile, *z* = −0.27). A physical exam of his back demonstrated an elevated right shoulder, a right thoracic prominence with significant rotation, and a left lumbar fullness on forward bending. Radiographic imaging showed a 42° right thoracic and 26° left thoracic curve, diminished vertebral body height of multiple mid and lower thoracic bodies, and concave endplates, reported as “suggestive of osteogenesis imperfecta or metabolic bone disease” (Figure 1R and S). Trio genome sequencing identified a homozygous nonsense variant in *SLC13A1*, NM_022444.4:c.144G>A p.(Trp48∗) with biallelic inheritance. Blood and urine sulfate levels could not be obtained.

### Bioinformatics and molecular modeling predicts disruption of SLC13A1

Bioinformatic assessments of *SLC13A1* variants are summarized in Supplemental Table 2. All *SLC13A1* variants demonstrated biallelic inheritance, consistent with autosomal recessive inheritance, and resulted in missense or nonsense changes. Homozygous missense variants were absent from the Genome Aggregation Database (gnomAD v4.1.0) for variants NM_022444.4: c.1343G>A p.(Gly448Asp), NM_022444.4:c.1547T>C p.(Leu516Pro) and NM_022444.4:c.1744T>C p.(Tyr582His), with heterozygous allele counts of 221, 7, and 3, respectively. By contrast, NM_022444.4:c.34C>T p.(Arg12∗) and NM_022444.4:c.709C>T p.(Arg237Cys) and NM_022444.4:c.1744T>C p.(Tyr582His) were observed in both the heterozygous and (rarely) in the homozygous state, with allele counts of 3884 heterozygous/7 homozygous; 3129 heterozygous/5 homozygous, respectively. In this context, it is important to note that the gnomAD v4.1.0 data set (GRCh38) spans 730,947 exome sequences and 76,215 genome sequences from unrelated individuals, of diverse ancestries, sequenced as part of various disease-specific and population genetic studies. All of the above *SLC13A1* missense or nonsense variants were predicted to be either pathogenic or damaging by 1 or more bioinformatic algorithms (Supplemental Table 2).

**Figure 2:**
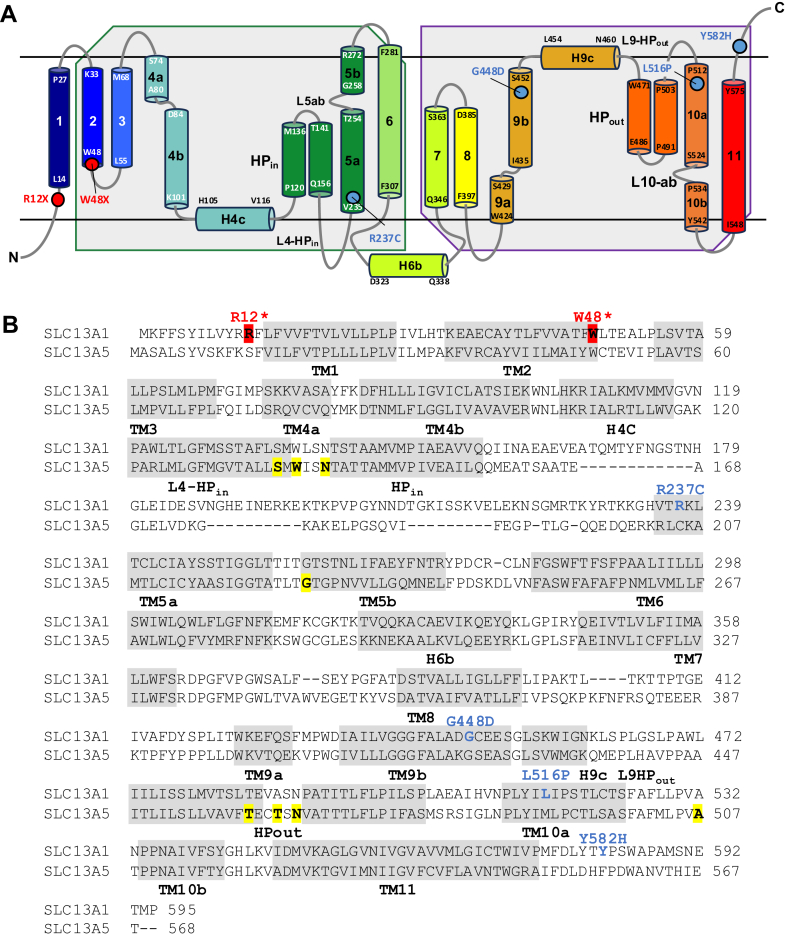
**Transmembrane topology of the sulphate transporter SLC13A1 and missense/nonsense variant locations.** A. Predicted transmembrane topology of SLC13A1 based on the cryo-electron microscopy structure of human SLC13A5 (adapted from Sauer et al^19^), indicating the relative positions of the p.(Arg12∗) (R12∗), p.(Trp48∗) (W48∗), p.(Arg237Cys) (R237), p.(Gly448Asp) (G448D), p.(Leu516Pro) (L516P), and p.(Tyr582His) (Y582H) variants. The beginning and end of each helix are numbered. Shaded regions indicate the inverted repeats of the protein. B. Alignment of SLC13A1 and SLC13A5 sequences. The position of nonsense variants p.(Arg12∗) and p.(Trp48∗) (red shading), and missense variants p.(Arg237Cys), p.(Gly448Asp), p.(Leu516Pro), and p.(Tyr582His) (blue shading) in SLC13A1 are highlighted. Predicted Na^+^-binding residues in SLC13A5 (Na1+: Ser136, Trp138, Asn141, and Gly226; Na2+: Thr460, Thr463, Asn465, and Ala507) are indicated by yellow shading. The scaffold domain is formed by transmembrane (TM) α-helices TMs 1-4 and 7-9, whereas the transport domain consists of TMs 5, 6, 10, and 11, and the helix hairpins HPin and HPout.

Because no experimentally determined 3D structures for human SLC13A1 are currently available, we used SwissModel to construct a homology model based on the structure^19^ of a related family member Na^+^-coupled citrate transporter, SLC13A5, in the inward-facing confirmation. This model had a strong qualitative model energy analysis score of 0.68 (range 0-1, with 1 being good) for the transmembrane protein, with lower confidence in the intracellular domain. The impacts of SLC13A1 missense substitutions on inter- and intramolecular interactions were based on their predicted locations in the protein model, which also predicts likely SLC13A1 transmembrane structure (Figure 2), using the ChimeraX molecular graphics system.^20^ Missense variants were modeled using the Swapaa command and the Dunbrack backbone-dependent rotamer library.^21^

**Figure 3:**
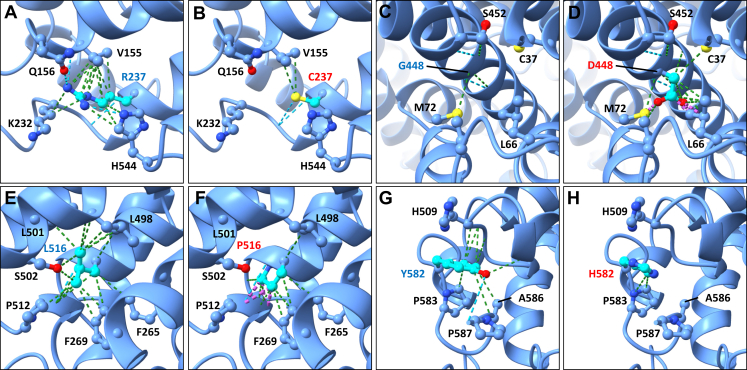
**Molecular modeling of human SLC13A1 variants.** A. Residue Arg237 (R237) is positioned at the intracellular end of transmembrane domain 5 (TM5), and the amphipathic side chain forms H-bonds with Q156 and H544 and contacts with V155, K232, and H544 as well. B. For substitution p.(Arg237Cys) (R237C), almost all contacts and H-bonds are lost, and a reactive cysteine is introduced. C. Residue G448 is located in the middle of TM9, where it makes minimal contact with M72 and S452. D. By contrast, substitution p.(Gly448Asp) (G448D) introduces a new contact with C37 in TM2 and significant clashes with L66 and M72. E. Residue Leu516 (L516) is located in TM10 and forms numerous contacts with F265 and F269 in TM5 and L498, L501, S502, and P512 in TM10. F. Substitution p.(Leu516Pro) (L516P) removes most contacts and introduces clashes with the backbone of the TM10 helix at P512 and L513. G. Tyr582 (Y582) is positioned within the extracellular C terminus of SLC13A1 and makes contacts with the P583 and A586 side chains and the backbone at G506, H509, and A586 and forms an H-bond with P587. H. Substitution p.(Tyr582His) (Y582H) removes all contacts and H-bonds other than with P583.

Residue Arg237 (R237) is located at the intracellular end of transmembrane domain 5 (TM5), where it is predicted to form hydrogen bonds (H-bonds) with Q156 and H544, and numerous contacts with V155, K232, and H544 (Figure 3A). TM5 is involved in conformational changes required for proper transport function, and disruptions affecting the movement of this helix may impede solute transport.^24^ For the p.(Arg237Cys) substitution (R237C), the cysteine residue is predicted to lose almost all contacts and H-bonds, preserving only 2 contacts with V155 while forming a new H-bond with the backbone at G233 (Figure 3B). Residue Gly448 is located in the middle of TM9, where it makes minimal contact with M72 and S452 (Figure 3C). TM9 is important in transducing conformational changes in the related Na^+^-coupled dicarboxylate transporter, SLC13A2.^25^ The p.(Gly448Asp) substitution (G448D) is predicted to introduce a new contact with C37 in TM2, and introduce significant clashes with L66 and M72 (Figure 3D). These clashes may disrupt the proper function of TM9 and prevent proper sulfate uptake. Residue Leu516 is located within TM10, where it forms numerous contacts with residues of neighboring helices including F265 and F269 in TM5 and L498, L501, S502, and P512 in TM10 (Figure 3E). The p.(Leu516Pro) substitution (L516P) is predicted to remove most of these contacts and introduce clashes with the backbone of the helix at P512 and L513 (Figure 3F). Prolines are also known to introduce kinks in helices and, as such, may disrupt the structure of this region, which forms part of the ligand recognition pocket. Lastly, residue Tyr582 is part of the extracellular C terminus of SLC13A1 and makes contacts with the side chains of P583 and A586 and backbone at G506, H509, and A586 and an H-bond with P587 (Figure 3G). The p.(Tyr582His) substitution (Y582H) is predicted to remove all contacts and H-bonds other than with P583 (Figure 3H).

### Functional analysis of *SLC13A1* sequence variants demonstrate complete loss of sulfate transport activity

**Figure 4:**
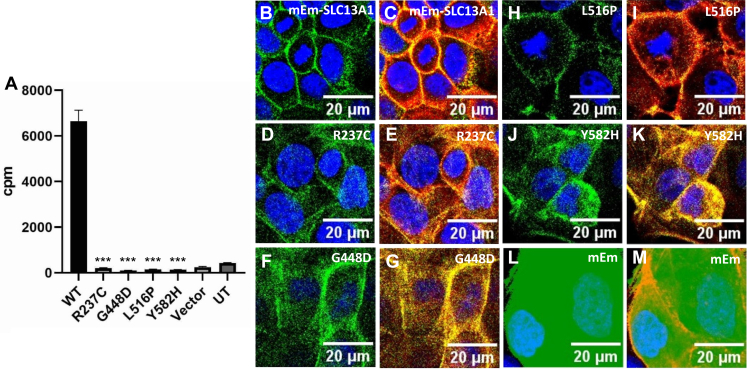
**Sulfate transport function and subcellular location of mEmerald-tagged SLC13A1 and missense variants.** A. Sulfate transport function of SLC13A1 control and variant proteins. Radiotracer [^35^S]-sulfate uptake into cultured MDCK cells stably transfected with pmEmerald vector containing wild-type SLC13A1 (WT), p.(R237C), p.(G448D), p.(L516P), p.(Y582H) mutant constructs, or empty pEmerald (Vector). UT represents untransfected MDCK cells. Mean ± SD. Data are representative of 3 separate experiments. Sulfate uptake for all SLC13A1 missense variants was significantly lower (∗∗∗ *P* < .0001) compared with control SLC13A1 using one-way ANOVA followed by a Dunnett’s multiple comparison test. B-M. Expression of mEmerald-tagged SLC13A1 proteins in MDCK cells. mEmerald fluorescence (green), nucleus (blue), actin (red), and overlapping mEmerald and actin (yellow, right panels). SLC13A1 control (B and C) and variant p.(R237C) (D and E), p.(G448D) (F and G), p.(L516P) (H and I), and p.(Y582H) (J and K) protein sequences. MDCK cells expressing mEmerald only (L and M).

Stably transfected MDCK cell lines were used to compare [^35^S]-sulfate transport capacity of control and variant SLC13A1 proteins. All 4 amino acid sequence substitutions, p.(Arg237Cys), p.(Gly448Asp), p.(Leu516Pro), and p.(Tyr582His), led to complete loss of sulfate transport activity because no detectable [^35^S]-sulfate transport was present for mutants compared with the control wild-type mEmerald-SLC13A1 construct (Figure 4A). The plasma membrane expression of each variant was examined using confocal microscopy and was similar to control mEmerald-SLC13A1 (Figure 4B-M), suggesting that these amino acid substitutions affect sulfate transport but do not appear to completely disrupt trafficking to the plasma membrane. Unlike the related sulfate transporter SLC13A4, which shows apical membrane expression,^23^ mEmerald-tagged SLC13A1 did not appear to discriminate between apical and basolateral membranes in MDCK cells.

## Discussion

To our knowledge, this is the first functional and phenotypic characterization of *SLC13A1* variants identified in a cohort of children with skeletal dysplasia and biallelic variants in *SLC13A1*. This genetic disorder is associated with a measurable biomarker in the blood (hyposulfatemia) and urine (hypersulfuria) that ultimately results in abnormal cartilage and bone development and possibly other clinical phenotypes that have yet to be fully recognized.

Sulfate is the fourth most abundant anion in human blood, with plasma levels between 0.3 and 0.5 mM in normal adults.^1^^,^^2^ As a divalent anion, sulfate cannot passively cross cell membranes, requiring all cells to express membrane sulfate transporters to maintain optimal intracellular concentrations of sulfate to allow for relevant metabolic processes to occur.^2^ *SLC13A1* encodes the apical membrane, sodium-sulfate cotransporter expressed primarily in the kidney and intestine where it mediates sulfate (re)absorption and maintenance of circulating sulfate levels to meet sulfate demands of other tissues.^1^, ^2^, ^3^, ^4^ As a result, genetic variants that result in complete loss and/or decreased activity of the SLC13A1 transporter lead to reduced plasma sulfate levels and increased urinary sulfate excretion.

This concept is demonstrated by sulfate measurements in proband 1 and proband 2. The urinary sulfate excretion in proband 1 compound heterozygous for p.(Arg12∗) and p.(Gly448Asp) was markedly elevated at 10.96 (ref 0.5-6.0). The plasma sulfate level in proband 2 with homozygous p.(Tyr582His) was proportionately reduced compared with reported individuals with heterozygous *SLC13A1* nonsense variants (0.107 vs 0.25 mM)^9^ and similar to the level reported in a child homozygous for the p.(Arg12∗) variant (0.065 mM).^17^ These in vivo studies are consistent with these missense variants in *SLC13A1* representing LOF alleles with similar effect size to that of *SLC13A1* nonsense variants. The bioinformatic modeling and in vitro functional studies performed in this study further support the notion that all *SLC13A1* variants in this cohort result in complete LOF, including the missense variants, p.(Arg237Cys), p.(Gly448Asp), p.(Leu516Pro), and p.(Tyr582His), which demonstrated similar reduction of [^35^S]-sulfate uptake into cultured MDCK cells compared with nonsense variants (Figure 4A) despite evidence of localization to the plasma membrane (Figure 4B-M).

The overt clinical phenotypes demonstrated in this cohort of individuals with biallelic LOF variants in *SLC13A1* and previously reported in *Slc13a1*-null mammals, demonstrate the essential role of this gene, and the developmental importance of maintaining adequate intracellular sulfate levels.^10^^,^^12^, ^13^, ^14^, ^15^, ^16^ Differences in bone and cartilage development appear to be the most apparent phenotype in individuals with biallelic *SLC13A1* LOF variants because this was the primary indication for clinical evaluation for all individuals in this cohort. However, Neff et al^16^ reported that *SLC13A1* is not expressed in cartilage or bone and concluded that the osteochondrodysplasia that results from the deletion of *SLC13A1* was secondary to the underlying metabolic disorder, which likely affects other systems, resulting in phenotypes that have yet to be described clinically. This concept is supported by detailed phenotyping of the *Slc13a1* knockout mouse by Dawson et al,^10^^,^^12^, ^13^, ^14^ demonstrating hyposulfatemia and impaired growth, along with behavioral differences, seizures, endocrine abnormalities, and reduced fertility. Proband 5 with homozygous p.(Trp48∗) exhibited a neurodevelopmental phenotype (Table 1), whereas a previously reported child with homozygous p.(Arg12∗) had autistic features.^17^ Whether *SLC13A1* LOF variants or possibly only nonsense variants, are associated with a neurodevelopmental phenotype will require identification and evaluation of additional individuals.

There are several other IMDs in which disturbed sulfate homeostasis is the underlying mechanism of disease (Supplemental Table 3). Biallelic LOF variants in *SLC26A2*, which encodes the predominant sulfate transporter in cartilage and bone,^26^^,^^27^ cause a spectrum of osteochondrodysplasias, including diastrophic dysplasia (OMIM 222600). Additionally, disruptions in sulfate-related pathways, such as glutathione metabolism, have been associated with spondylometapheseal dysplasia (OMIM 250220).^28^ Despite the importance of sulfate for numerous cellular and metabolic processes in humans, it is not routinely measured for clinical purposes.^1^^,^^3^

As with many IMDs, identification and recognition of the underlying biochemical disturbance provides insight regarding management and treatment. Metabolism of organic sulfur compounds, including sulfur-containing amino acids methionine and cysteine, supply over half of the daily intake of sulfate, whereas the remainder is supplied from ingestion of inorganic sulfate in drinking water and food.^29^ Monti et al^30^ found that prenatal administration of N-acetyl-L-cysteine (NAC) to mouse fetuses with *SLC26A2*-associated diastrophic dysplasia via the drinking water of the pregnant females ameliorated the postnatal skeletal phenotype that results from the limited extracellular sulfate supply and subsequently reduced sulfation of macromolecules in the disorder. The use of oral sulfate supplementation, either directly or via other sulfate sources, such as NAC, to saturate other collateral sulfate transporters and any residual SLC13A1 function is an approach that warrants further evaluation in humans.

The close biochemical relationship between sulfate, NAC, and glutathione is one that likely has medical and therapeutic implications for *SLC13A1*-associated disease and other IMDs of disturbed sulfate homeostasis. NAC is metabolized to cysteine, which can serve as a sulfate donor for sulfation of molecules, and a precursor for glutathione synthesis.^31^ For these reasons, oral or intravenous NAC is used in cases of acetaminophen-induced hepatotoxicity because it likely protects the liver by promoting the nontoxic metabolism of acetaminophen via sulfation, maintenance and/or restoration of glutathione levels, and reduction of the hepatotoxic intermediate, *N*-acetyl-p-benzoquinone imine.^32^ There is great interindividual variation in susceptibility to acetaminophen toxicity; acetaminophen-induced hepatotoxicity has been reported in adults taking the recommended daily maximum dose of 4 g/day or less,^33^^,^^34^ a treatment regimen prescribed to approximately 6% of US adults.^35^ Acetaminophen decreases serum sulfate concentrations in normal adults because of its partial metabolism via sulfation,^36^ making *SLC13A1* an obvious pharmacogenetic candidate, further supported by disruption of *Slc13a1* in mice, leading to hyposulfatemia and enhanced acetaminophen-induced hepatotoxicity resulting from decreased sulfation capacity to metabolize acetaminophen.^11^ For these reasons, caution should be taken in the administration and dosing of acetaminophen and other medications metabolized via sulfation to individuals with biallelic *SLC13A1* LOF variants until the full clinical phenotype is better understood.

Evidence of clinical phenotypes in individuals with heterozygous *SLC13A1* LOF variants further highlights the importance of sulfate in human disease and broader clinical impact of altered sulfate homeostasis.^9^^,^^37^^,^^38^ A genome-wide association study of intervertebral disc disorder performed by Bjornsdottir et al^38^ in 2022 found *SLC13A1* nonsense variants p.(Arg12∗) and p.(Trp48∗) conferred the largest risk effect (LOF burden OR = 1.44, *P* = 3.1 × 10^−11^). In addition, the previously reported association between *SLC13A1* nonsense variants and reduced serum sulfate levels^9^ was replicated, demonstrating a 32.6% reduction of serum sulfate among individuals heterozygous for p.(Arg12∗).^38^ These findings may have clinical implications for the parents and relatives of the probands in this study. Outside of monogenic disorders of sulfate homeostasis, sulfate biology likely plays an important role in many common skeletal and cartilage diseases with implications regarding treatment. For example, glucosamine sulfate is an oral supplement widely used to treat symptoms of osteoarthritis. Glucosamine sulfate’s mechanism of action was thought to be through increased glucosamine concentrations in the joint space, leading to stimulation of articular cartilage glycosaminoglycan synthesis. However, this was deemed unlikely by Hoffer et al^41^ because even large doses of glucosamine sulfate have no effect on serum glucosamine levels.^39^, ^40^, ^41^ In contrast, additional studies demonstrated that oral glucosamine sulfate increases serum sulfate concentrations in humans, notably an effect reversed by concurrent injection of acetaminophen; thus, it is more likely that sulfate, rather than glucosamine, is mediating the therapeutic effects of glucosamine sulfate therapy in osteoarthritis.^41^

In conclusion, biallelic LOF variants in *SLC13A1* are a novel cause of skeletal phenotypes in humans that result from impaired sulfate transport and hyposulfatemia. In 2 of the 5 cases, a laboratory offering sulfate measurements was accessible to clinicians to aid in the clinical interpretation of *SLC13A1* variants. This work provides strong support for the addition of *SLC13A1* to short stature, scoliosis, and skeletal dysplasia gene panels, and development of clinical sulfate quantification assays in more laboratories around the world to allow for biochemical phenotyping of individuals with *SLC13A1* variants, better understand the role of sulfate in human disease, and inform future treatment options.

## Data Availability

All data described in this study are provided within the article and supplemental materials. This study did not generate data sets or code. All methods are available on request. Additional deidentified clinical data are available upon request to the corresponding authors.

## Conflict of Interest

The authors declare no conflicts of interest.
