# Supplementary material for: Biallelic SLC13A1 loss-of-function variants result in impaired sulfate transport and skeletal phenotypes, including short stature, scoliosis, and skeletal dysplasia
Source: Genet Med Open. 2024 Dec 26;3:101958. doi: 10.1016/j.gimo.2024.101958 (PMC11803892; doi:10.1016/j.gimo.2024.101958)
Supplement: Supplemental Figures [file mmc1.pptx]

## Slide 1
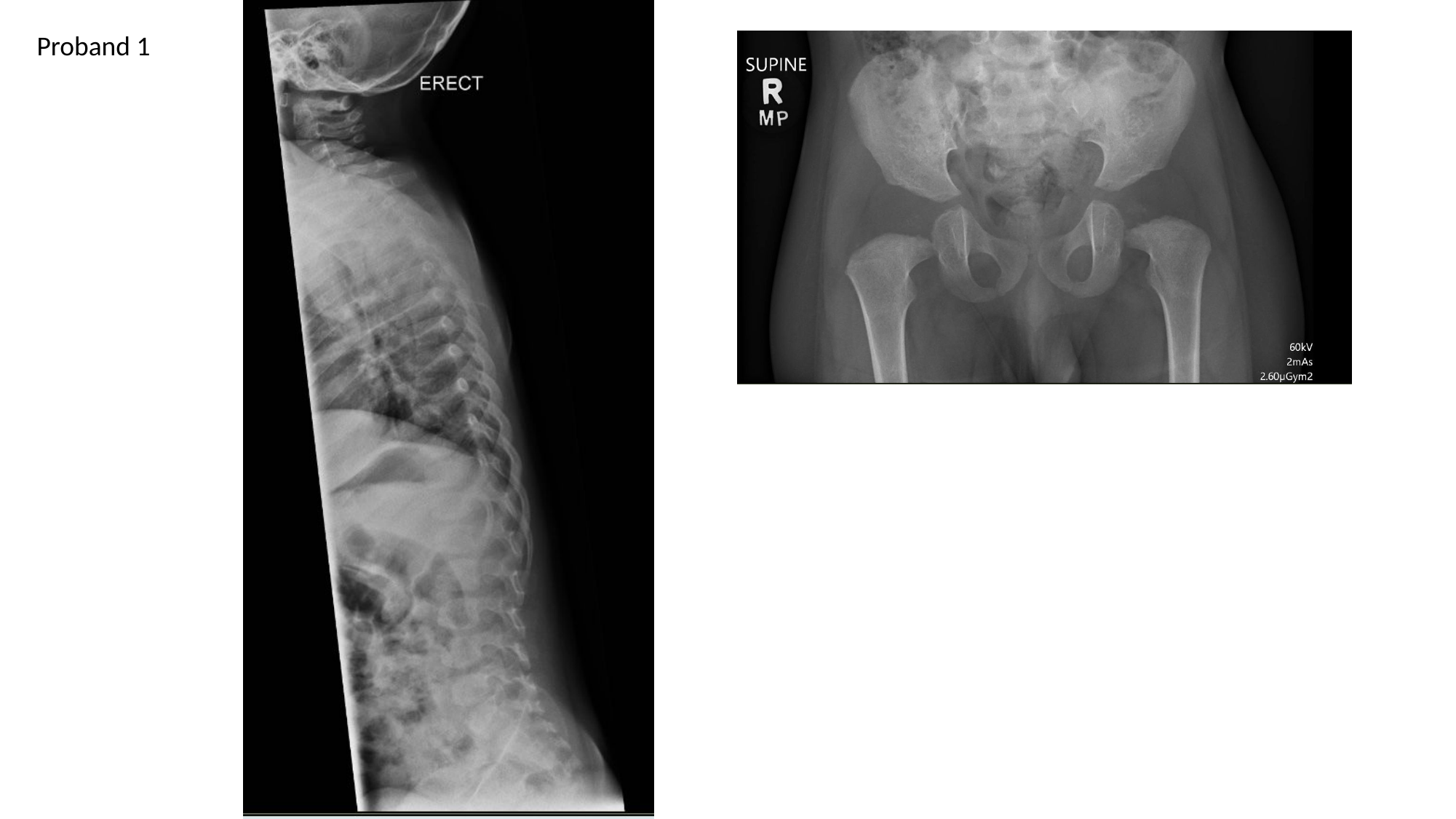

Proband 1

## Slide 2
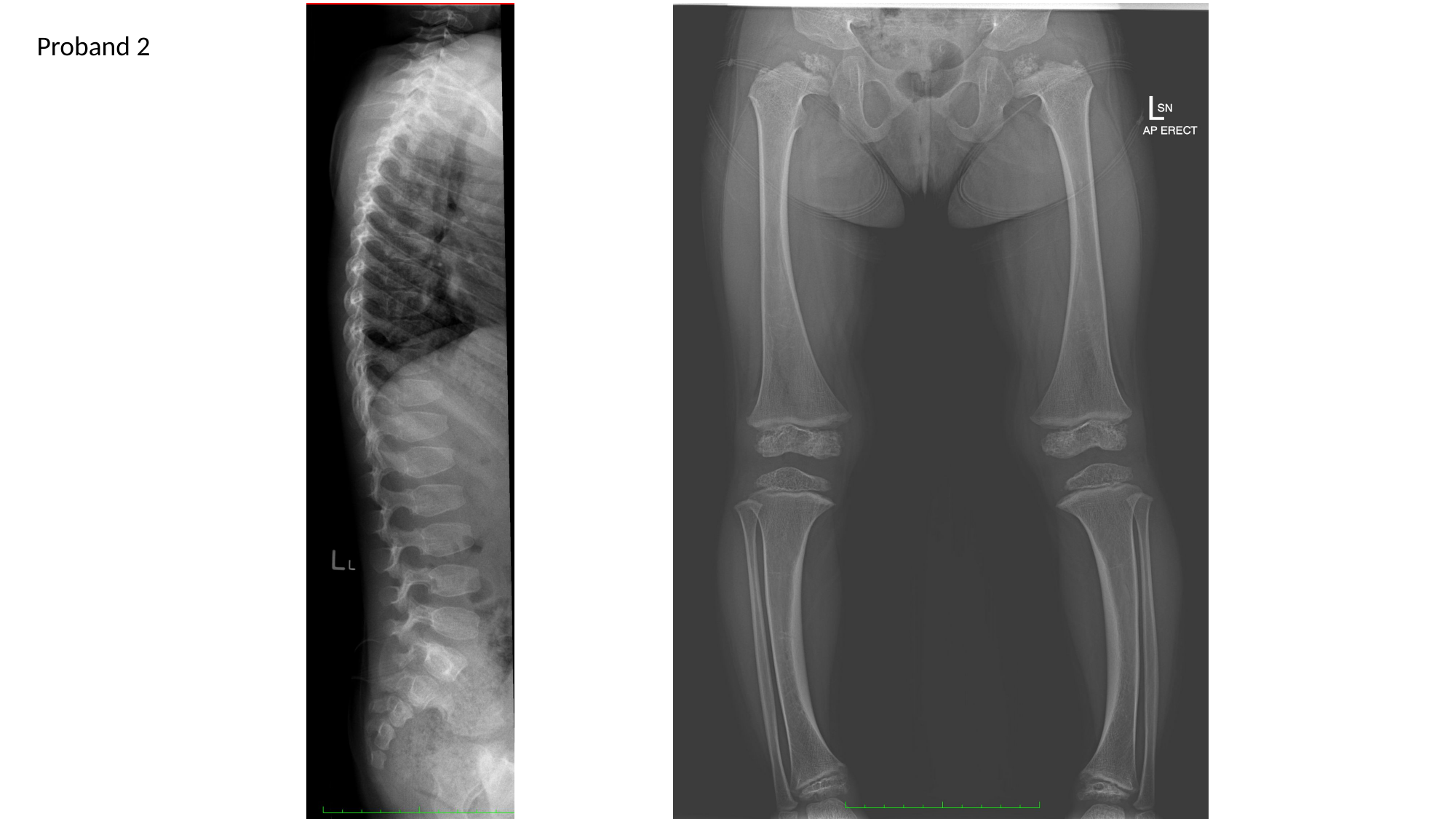

Proband 2

## Slide 3
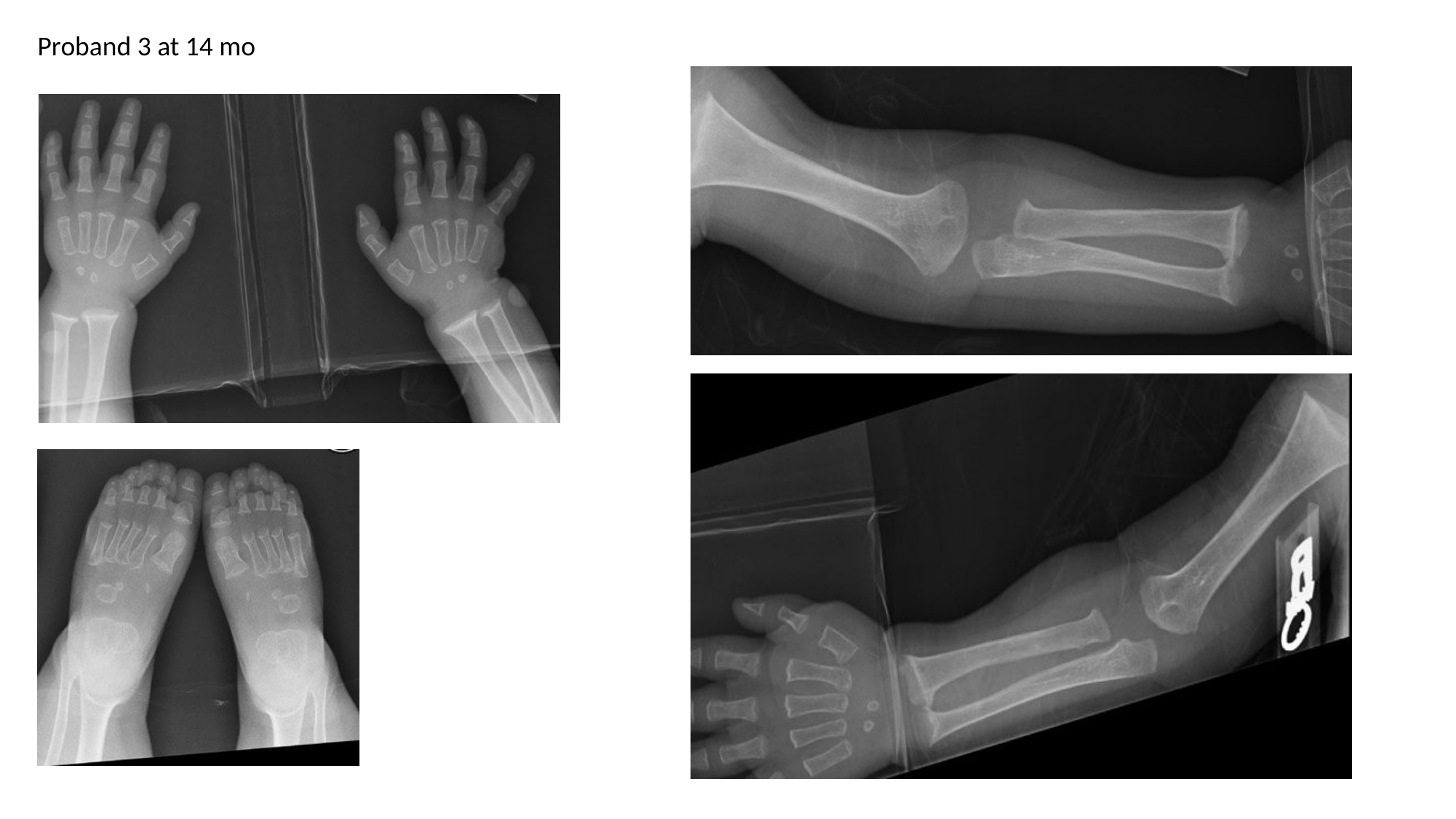

Proband 3 at 14 mo

## Slide 4
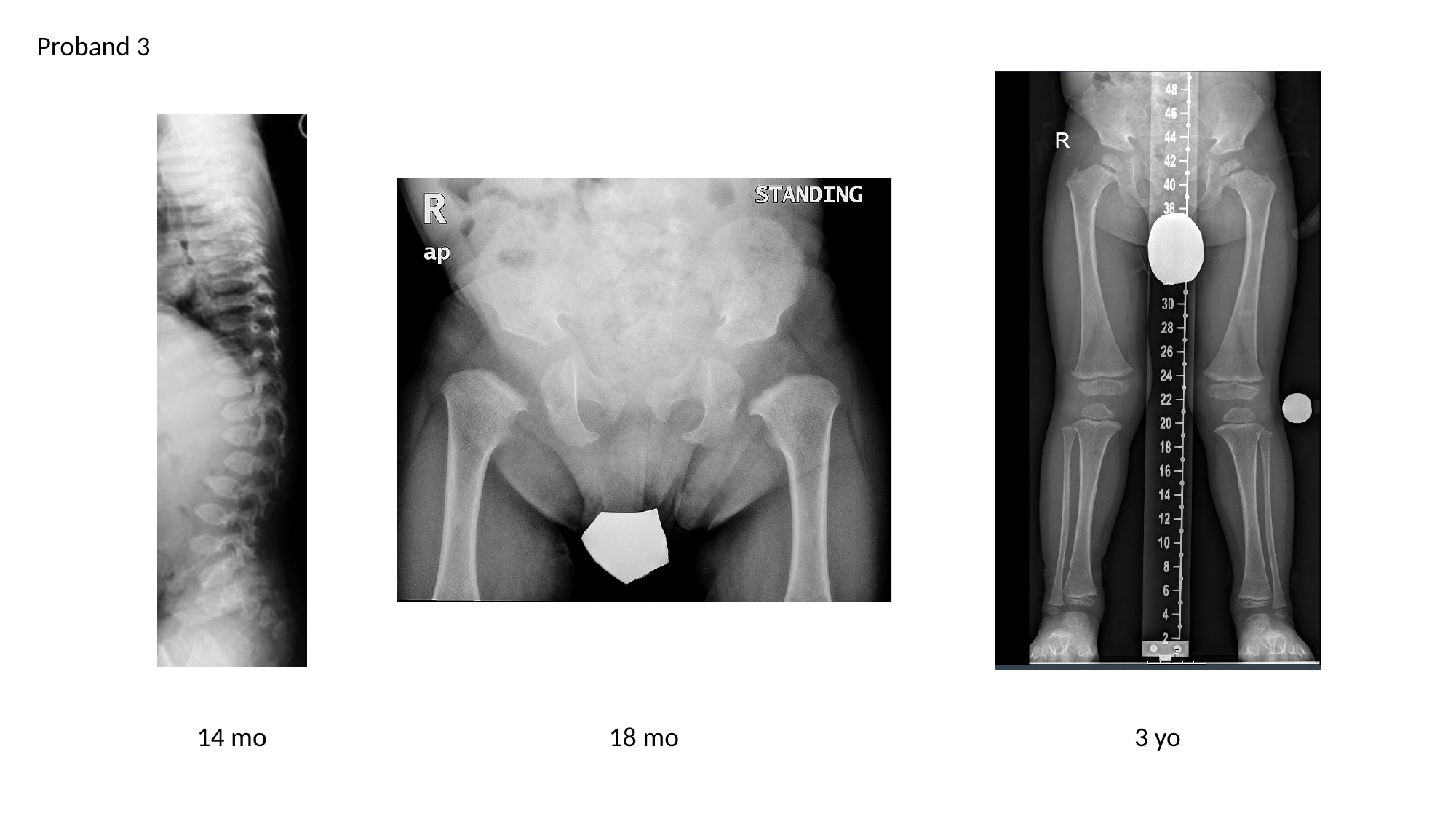

Proband 3
14 mo
18 mo
3 yo

## Slide 5
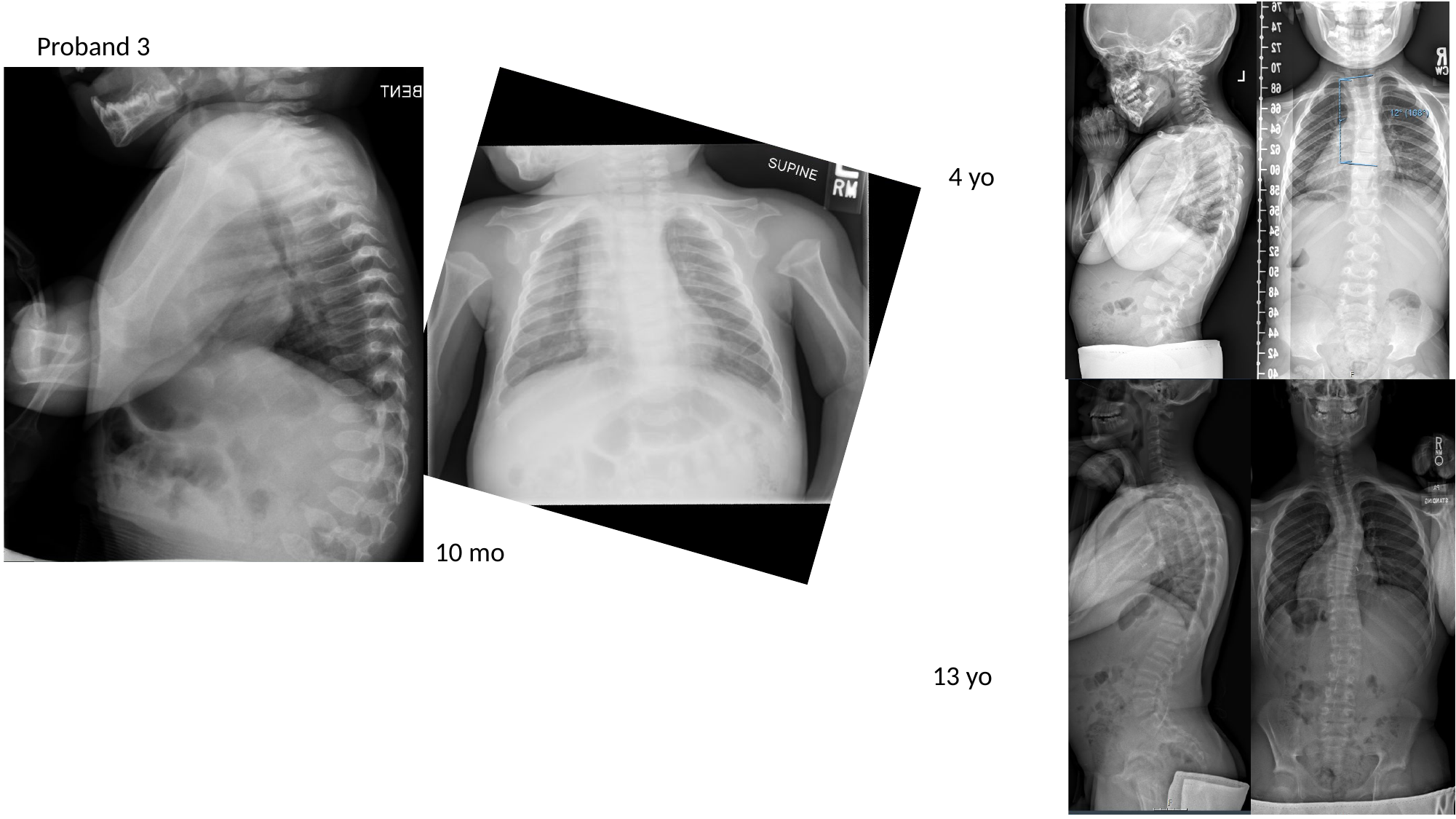

Proband 3
4 yo
10 mo
13 yo

## Slide 6
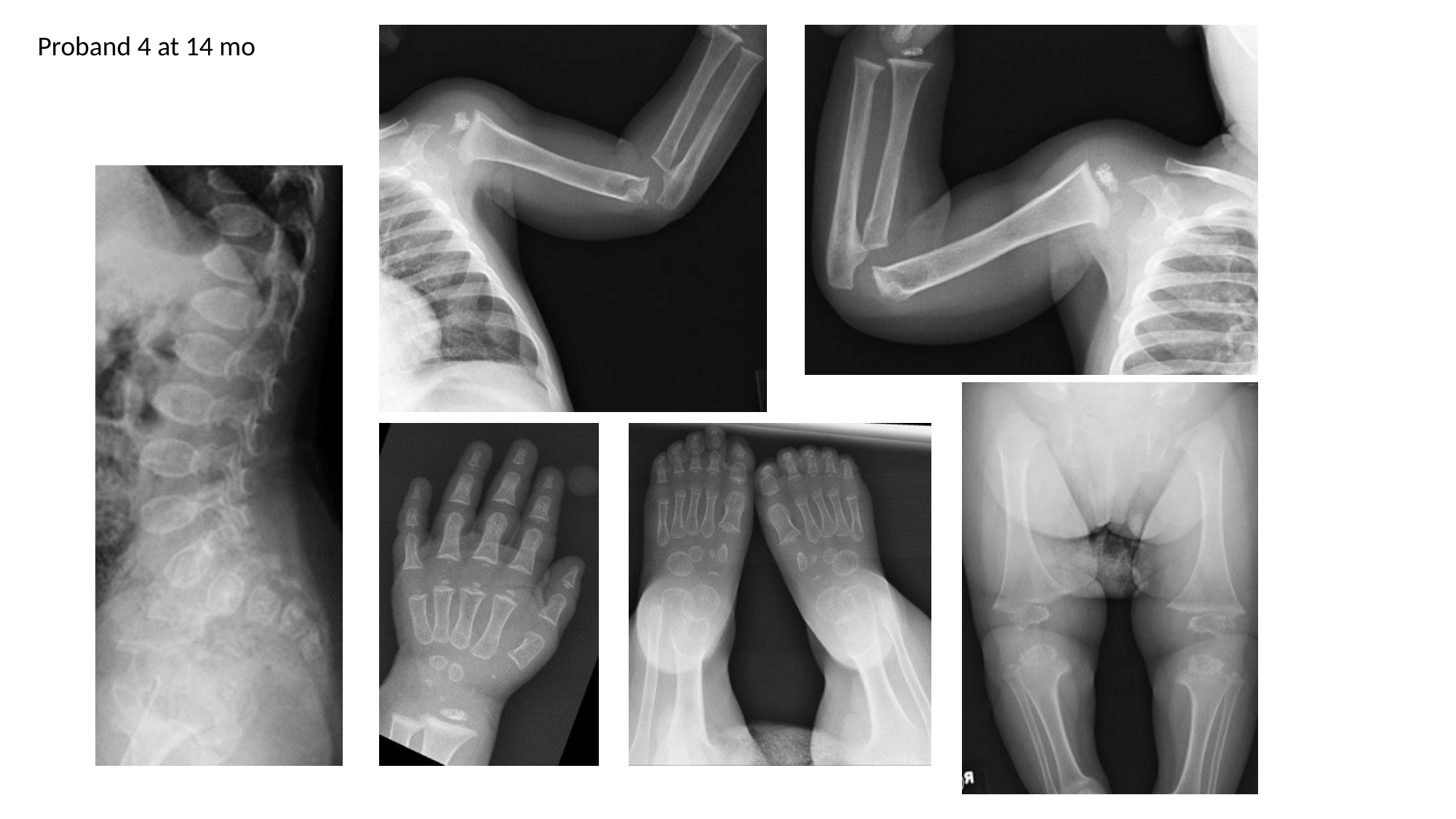

Proband 4 at 14 mo

## Slide 7
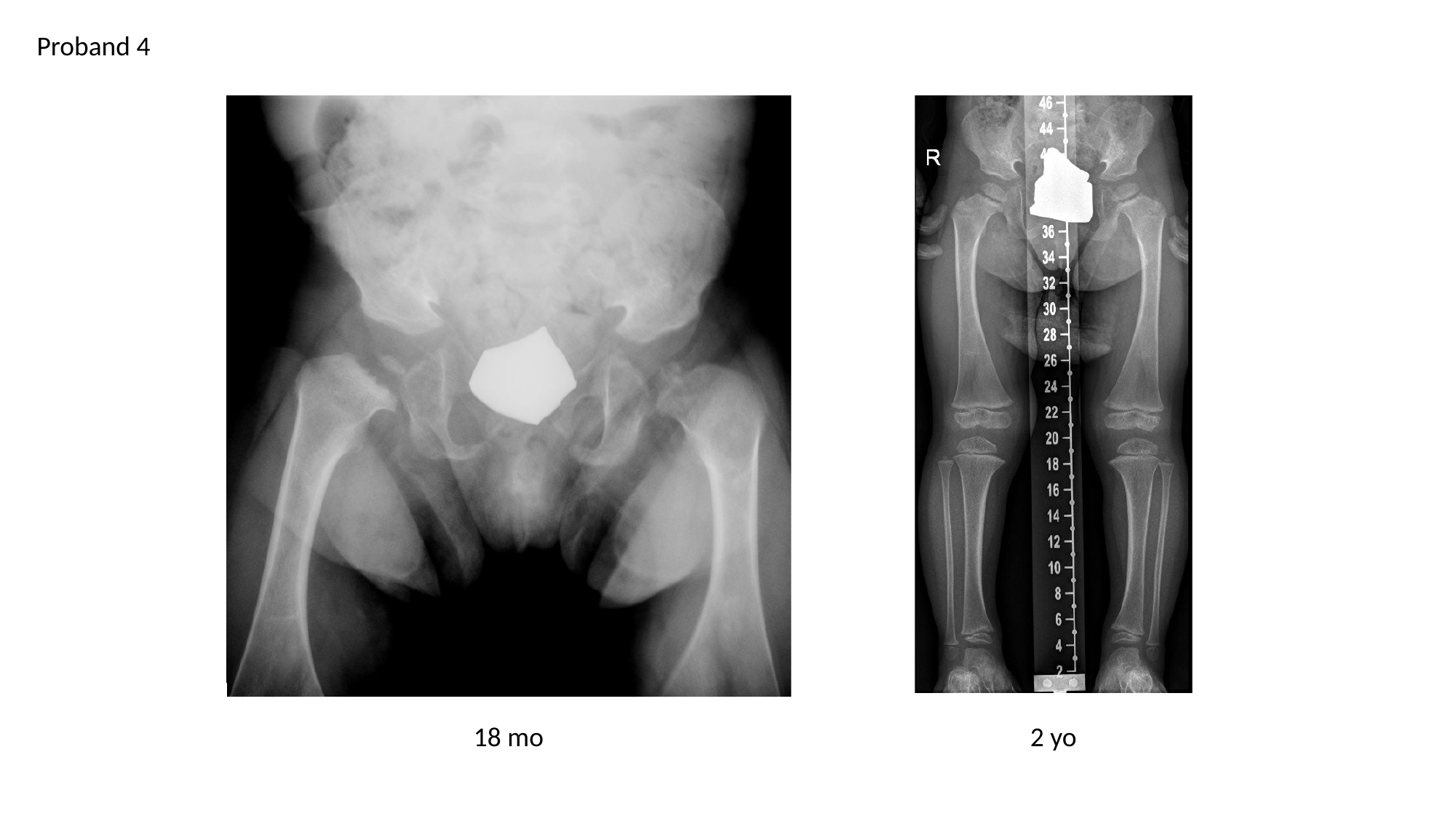

Proband 4
18 mo
2 yo

## Slide 8
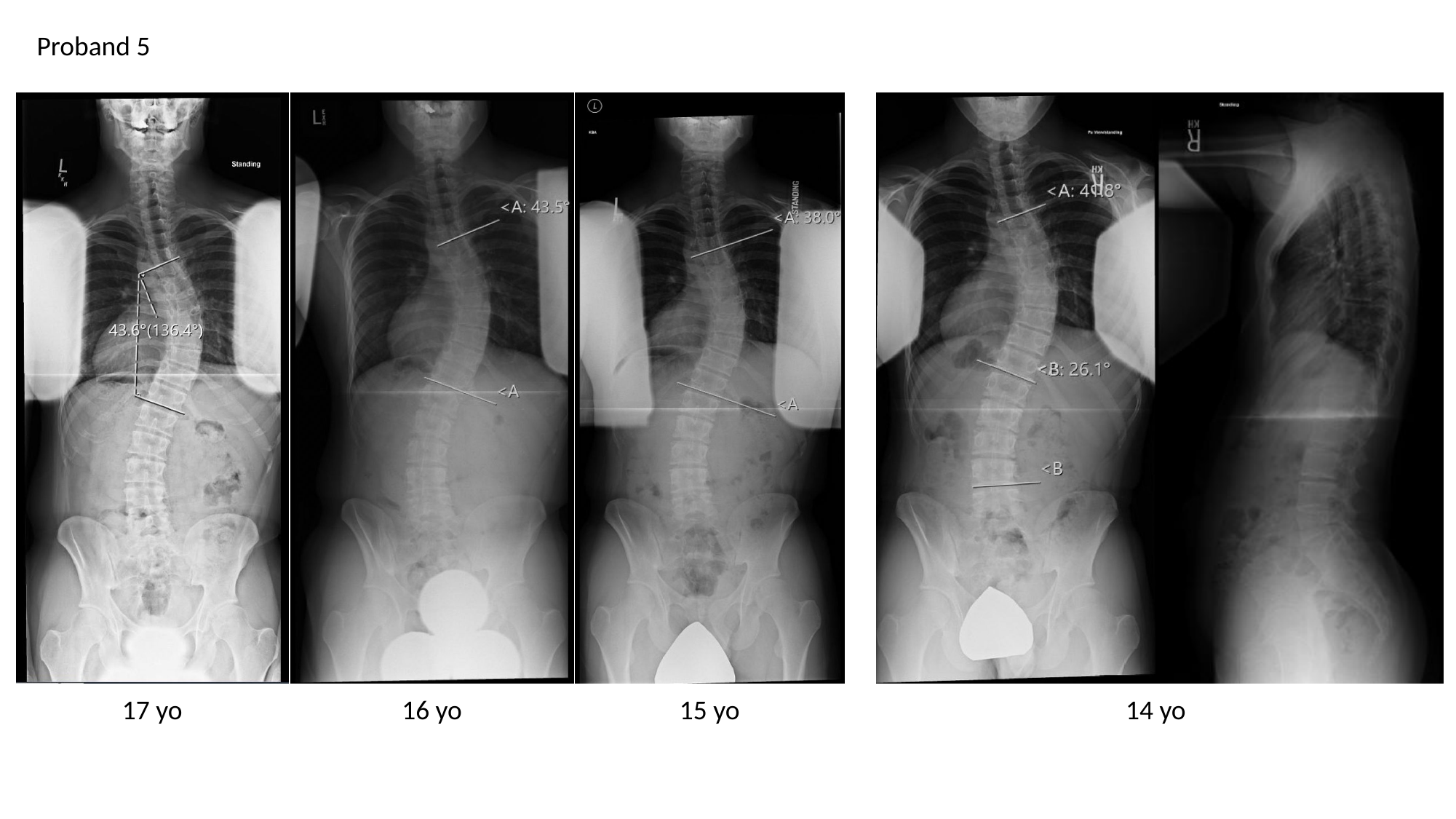

Proband 5
17 yo
16 yo
15 yo
14 yo

## Slide 9
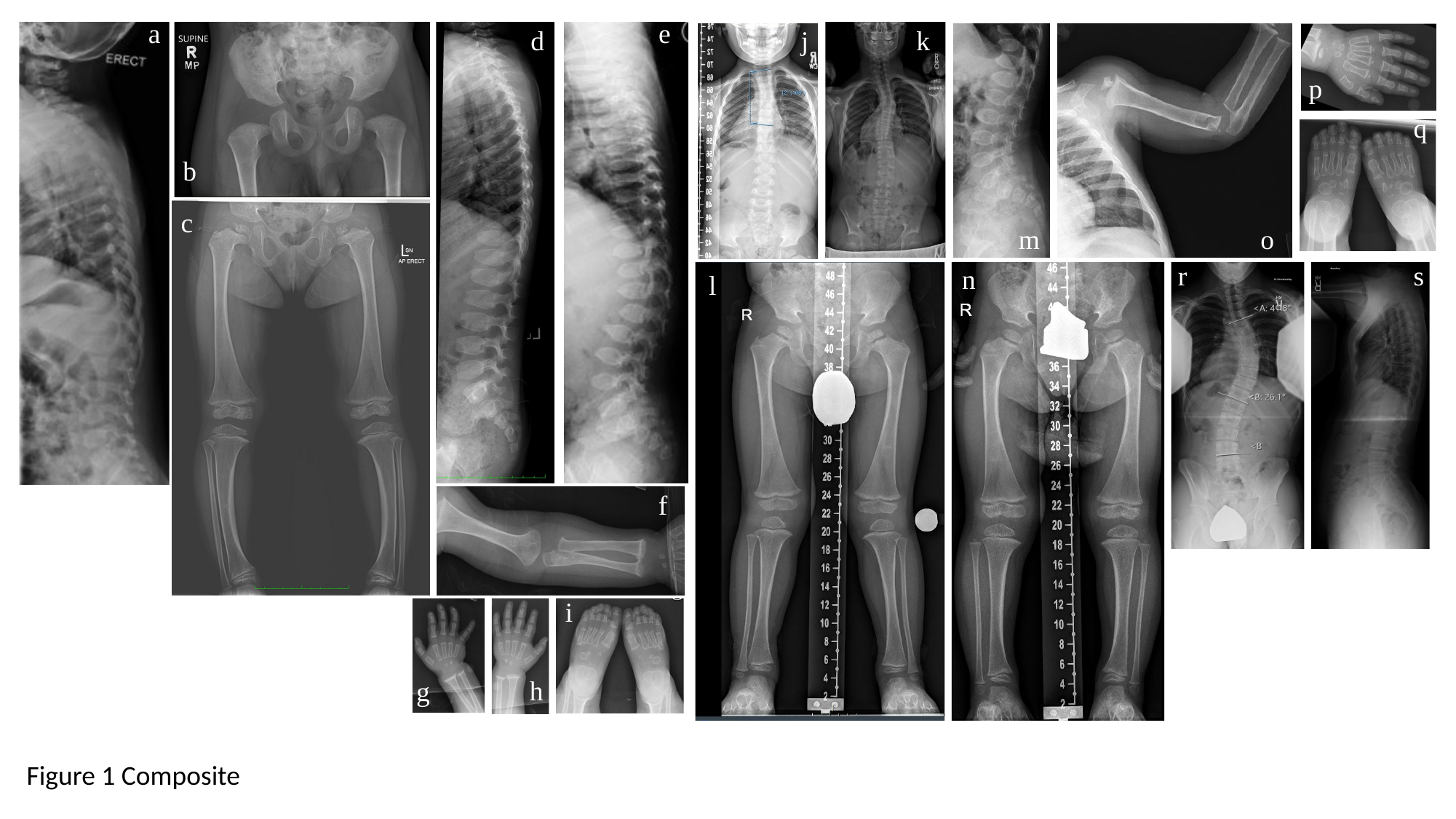

a
e
d
j
k
p
q
b
c
m
o
s
r
n
l
f
i
h
g
Figure 1 Composite
