# Supplementary material for: Biallelic SLC13A1 loss-of-function variants result in impaired sulfate transport and skeletal phenotypes, including short stature, scoliosis, and skeletal dysplasia
Source: Genet Med Open. 2024 Dec 26;3:101958. doi: 10.1016/j.gimo.2024.101958 (PMC11803892; doi:10.1016/j.gimo.2024.101958)
Supplement: Supplemental Tables [file mmc2.docx]

**SUPPLEMENTARY DATA**

**Biallelic *SLC13A1* loss-of-function variants result in impaired sulfate transport and skeletal phenotypes including short stature, scoliosis, and skeletal dysplasia**

| **Primer name** | **Sequence** | **Target** |
| --- | --- | --- |
| pmEm-C1F | gcgggcccgggatccaccggatctagataa | pmEmerald-C1 |
| pmEm-C1R | aagcttgagctcgagatctgagtccggact | pmEmerald-C1 |
| pmEm-SLC13A1F | agtccggactcagatctcgagctcaagcttcaatgaaattcttcagttacattctgg | SLC13A1 cDNA |
| pmEm-SLC13A1R | ttatctagatccggtggatcccgggcccgcttatggcatggtctcattactcata | SLC13A1 cDNA |
| SLC13A1-R237C1 | agaagggccacgtgacatgtaaacttacgtgtttgtg | SLC13A1 cDNA |
| SLC13A1-R237C2 | cacaaacacgtaagtttacatgtcacgtggcccttct | SLC13A1 cDNA |
| SLC13A1-G448D1 | ggtttgccctggcagatgattgtgaggagtctggatt | SLC13A1 cDNA |
| SLC13A1-G448D2 | aatccagactcctcacaactatctgccagggcaaacc | SLC13A1 cDNA |
| SLC13A1-L516P1 | tgaaccctctttatattccgataccttctactctgtg | SLC13A1 cDNA |
| SLC13A1-L516P2 | cacagagtagaaggtatcggaatataaagagggttca | SLC13A1 cDNA |
| SLC13A1-Y582H1 | tgtttgacctctacactcacccttcgtgggctcctgc | SLC13A1 cDNA |
| SLC13A1-Y582H2 | gcaggagcccacgaagggtgagtgtagaggtcaaaca | SLC13A1 cDNA |
| ColE1F | ggagcgaacgacctacaccgaactgagatacctacagcg | pmEmerald-C1 |
| ColE1R | cgctgtaggtatctcagttcggtgtaggtcgttcgctcc | pmEmerald-C1 |
| SLC13A1S1 | ctagaaattgatgaaagt | SLC13A1 cDNA |
| SLC13A1S2 | ggttttgctacagattca | SLC13A1 cDNA |

**Supplemental Table 1. PCR primers used for human *SLC13A1* constructs, mutagenesis and DNA sequencing.**

| **GRCh38/hg38 location** | NC_000002.7:  g.123199913 G>A | NC_000002.7:  g.123181057 C>T | NC_000002.7:  g.123169180 T>C | NC_000002.7:  g.123147262 G>A | NC_000002.7:  g.123123133 C>T | NC_000002.7:  g.123117574 A>G | NC_000002.7:  g.123115562 A>G |
| --- | --- | --- | --- | --- | --- | --- | --- |
| **HGVS cDNA** | c.34C>T | c.144G>A | c.521A>G | c.709C>T | c.1343G>A | c.1547T>C | c.1744T>C |
| **HGVS protein** | p.(Arg12*) | p.(Trp48*) | p.(Asn174Ser) | p.(Arg237Cys) | p.(Gly448Asp) | p.(Leu516Pro) | p.(Tyr582His) |
| **CADD** | **34** | **36** | 0.235 | **18.6** | **26** | **26.8** | **28.3** |
| **Clinpred** | - | - | 0.001 | 0.0348 | **0.9885** | **0.9885** | **0.9983** |
| **Polyphen-2** | - | - | 0.000/  Benign | 0.000/  Benign | **1.000/**  **Probably Damaging** | **1.000/**  **Probably Damaging** | **1.000/**  **Probably Damaging** |
| **REVEL** | - | - | 0.023 | 0.135 | 0.460 | 0.426 | **0.548** |
| **SIFT** | - | - | Tolerated | Tolerated | **Deleterious** | **Deleterious** | **Deleterious** |

**Supplemental Table 2. Summary of selected *in silico* scores for *SLC13A1* missense variants, using CADD^2^, ClinPred^3^, Polyphen-2^4^, REVEL^5^ and SIFT.^6^**

Genomic coordinates are from GRCh38/hg38 and cDNA and protein coordinates use *Homo sapiens* solute carrier family 13 member 1 (SLC13A1) transcript variant 1 (NM_022444.4 and NP_071889.2). Variant NM_022444.4:c.521A>G p.(Asn174Ser), which does not affect serum sulfate concentrations in the heterozygous or homozygous state,^7^ is shown as a comparator. With the exception of p.(Asn174Ser)*, SLC13A1* missense or nonsense variants were predicted to be either pathogenic or damaging by one or more software packages. CADD predicts a continuous phred-like score that ranges from 1 to 99, higher values indicating more deleterious cases. CADD cutoffs for deleteriousness (i.e. to identify potentially pathogenic variants)**^2^** were set between between 10 and 20. ClinPred and REVEL scores *lower* than 0.5 are defined as tolerated and *greater* than 0.5 as damaging. SIFT, which sorts intolerant from tolerant substitutions, classifies substitutions as tolerated or deleterious.

| **Protein Function** | **Gene** | **Loci** | **Phenotype/Syndrome** | **OMIM#** | **Inheritance** |
| --- | --- | --- | --- | --- | --- |
| Sulfate transporter | *SLC26A2* | *5q32* | Achondrogenesis Ib | 600972 | AR |
|  |  |  | Atelosteogenesis II | 256050 | AR |
|  |  |  | De la Chapelle dysplasia | 256050 | AR |
|  |  |  | Diastrophic dysplasia | 222600 | AR |
|  |  |  | Diastrophic dysplasia, broad bone-platyspondylic variant | 222600 | AR |
|  |  |  | Epiphyseal dysplasia, multiple, 4 | 226900 | AR |
|  | *SLC26A3* | *7q22.3-7q31.1* | Congenital secretory chloride diarrhea | 214700 | AR |
|  | *SLC26A8* | *6p21.31* | Spermatogenic failure 3 | 606766 | AD |
|  | *SLC13A1* | *7q31.32* | Spondyloepimetaphyseal dysplasia | TBD | AR |
| PAPS synthase | *PAPSS2* | *10q23.2-10q23.3* | Brachyolmia 4 with mild epiphyseal and metaphyseal changes | 612847 | AR |
| Sulfotransferase | *CHST3* | *10q22.1* | Spondyloepiphyseal dysplasia with congenital joint dislocations | 143095 | AR |
|  | *CHST6* | *16q23.1* | Macular corneal dystrophy | 217800 | AR |
|  | *CHST3* | *19q13.11* | Peeling skin syndrome 3 | 616265 | AR |
|  | *CHST11* | *12q23.3* | Osteochondrodysplasia, brachydactyly, and overlapping malformed digits | 618167 | AR |
|  | *CHST14* | *15q15.1* | Ehlers-Danlos syndrome, musculocontractural type 1 | 601776 | AR |
|  | *HS2ST1* | *1p22.3* | Neurofacioskeletal syndrome with or without renal agenesis | 619194 | AR |
|  | *HS6ST1* | *2q14.3* | Hypogonadotropic hypogonadism 15 with or without anosmia | 614880 | AD |
|  | *NDST1* | *5q33.1* | Intellectual developmental disorder, autosomal recessive 46 | 616116 | AR |
| Sulfatase | *ARSA* | *22q13.33* | Metachromatic leukodystrophy | 250100 | AR |
|  | *ARSB* | *5q14.1* | Mucopolysaccharidosis type VI (Maroteaux-Lamy) | 253200 | AR |
|  | *ARSE* | *Xp22.33* | Chondrodysplasia punctata | 302950 | XLR |
|  | *GALNS* | *16q24.3* | Mucopolysaccharidosis IVA (Morquio A) | 253000 | AR |
|  | *GNS* | *12q14.3* | Mucopolysaccharidosis type IIID | 252940 | AR |
|  | *IDS* | *Xq28* | Mucopolysaccharidosis II (Hunter) | 309900 | XLR |
|  | *SGSH* | *17q25.3* | Mucopolysaccharidisis type IIIA (Sanfilippo A) | 252900 | AR |
|  | *STS* | *Xp22.31* | Ichthyosis, X-linked | 308100 | XLR |
|  | *SUMF1* | *3p26.1* | Multiple sulfatase deficiency | 272200 | AR |
| Glutathione metabolism | *GPX4* | *19p13.3* | Spondylometaphyseal dysplasia, Sedaghatian type | 250220 | AR |

**Supplemental Table 3. Inherited metabolic disorders of perturbed sulfate homeostasis.** Modified from Dawson et al.^8^ *Abbreviations: OMIM, Online Mendelian Inheritance in Man; AR, autosomal recessive; AD, autosomal dominant; XLR, X-linked recessive.*

**Supplementary References**

1. Sauer DB, Song J, Wang B, et al. Structure and inhibition mechanism of the human citrate transporter NaCT. *Nature*. 2021;591(7848):157-161. https://doi.org/10.1038/s41586-021-03230-x
2. Rentzsch P, Witten D, Cooper GM, et al. CADD: predicting the deleteriousness of variants throughout the human genome. *Nucleic Acids Res*. 2019;47(D1): D886-D894. https://doi.org/10.1093/nar/gky1016
3. Alirezaie N, Kernohan KD, Hartley T, et al. ClinPred: prediction tool to identify disease-relevant nonsynonymous single-nucleotide variants. *Am J Hum Genet*. 2018;103(4):474-483. https://doi.org/10.1016/j.ajhg.2018.08.005
4. Adzhubei I, Jordan DM, Sunyaev SR. Predicting functional effect of human missense mutations using PolyPhen-2. *Curr Protoc Hum Genet*. 2013; Chapter 7:Unit 7.20. https://doi.org/10.1002/0471142905.hg0720s76
5. Ioannidis NM, Rothstein JH, Pejaver V, et al. REVEL: An ensemble method for predicting the pathogenicity of rare missense variants. *Am J Hum Genet*. 2016;99(4):877-885. https://doi.org/10.1016/j.ajhg.2016.08.016
6. Ng PC, Henikoff S. Predicting deleterious amino acid substitutions. *Genome Res.* 2001;11(5):863-874. https://doi.org/10.1101/gr.176601.
7. Tise CG, Perry JA, Anforth LE, et al. From genotype to phenotype: Nonsense variants in *SLC13A1* are associated with decreased serum sulfate and increased serum aminotransferases. G3 (Bethesda) 201; 6(9):2909-2918. https://doi.org/10.1534/g3.116.0329797
8. Dawson PA. Role of sulphate in development. *Reproduction*. 2013;146(3):R81-89. https://doi.org/10.1530/rep-13-0056

**Supplementary Web Resources**

CADD: Combined Annotation Dependent Depletion Score: http://cadd.gs.washington.edu/

ClinPred: https://sites.google.com/site/clinpred/

gnomAD: https://gnomad.broadinstitute.org/

Polyphen-2: http://genetics.bwh.harvard.edu/pph2/

RCSB PDB: http://www.rcsb.org/pdb/

REVEL: https://sites.google.com/site/revelgenomics/

SIFT: https://sift.bii.a-star.edu.sg/
